# Frequency, Risk Factors, and Adverse Fetomaternal Outcomes of Placenta Previa in Northern Tanzania

**DOI:** 10.1155/2017/5936309

**Published:** 2017-02-21

**Authors:** Elizabeth Eliet Senkoro, Amasha H. Mwanamsangu, Fransisca Seraphin Chuwa, Sia Emmanuel Msuya, Oresta Peter Mnali, Benjamin G. Brown, Michael Johnson Mahande

**Affiliations:** ^1^Kilimanjaro Christian Medical University College, Moshi, Tanzania; ^2^Department of Epidemiology & Biostatistics, Institute of Public Health, Kilimanjaro Christian Medical University College, Moshi, Tanzania; ^3^Department of Community Health, Institute of Public Health, Kilimanjaro Christian Medical University College, Moshi, Tanzania; ^4^Department of Global Health, Weill Cornell Medical College, New York, NY, USA

## Abstract

*Background and Objective*. Placenta previa (PP) is a potential risk factor for obstetric hemorrhage, which is a major cause of fetomaternal morbidity and mortality in developing countries. This study aimed to determine frequency, risk factors, and adverse fetomaternal outcomes of placenta previa in Northern Tanzania.* Methodology*. A retrospective cohort study was conducted using maternally-linked data from Kilimanjaro Christian Medical Centre birth registry spanning 2000 to 2015. All women who gave birth to singleton infants were studied. Adjusted odds ratios (ORs) with 95% confidence intervals for risk factors and adverse fetomaternal outcomes associated with PP were estimated in multivariable logistic regression models.* Result*. A total of 47,686 singleton deliveries were analyzed. Of these, the frequency of PP was 0.6%. Notable significant risk factors for PP included gynecological diseases, alcohol consumption during pregnancy, malpresentation, and gravidity ≥5. Adverse maternal outcomes were postpartum haemorrhage, antepartum haemorrhage, and Caesarean delivery. PP increased odds of fetal Malpresentation and early neonatal death.* Conclusion.* The prevalence of PP was comparable to that found in past research. Multiple independent risk factors were identified. PP was found to have associations with several adverse fetomaternal outcomes. Early identification of women at risk of PP may help clinicians prevent such complications.

## 1. Introduction

Placenta previa is an obstetric complication characterized by placental implantation into the lower segment of the uterine wall, covering whole (major) or part (minor) of the cervix [1]. It complicates 0.4% of pregnancies at term [2]. Placenta previa usually presents with painless vaginal bleeding in the late second or early third trimester. It is diagnosed on ultrasound during the second trimester or incidentally during an operation.

A study in Uganda reported an association between placenta previa and severe obstetric hemorrhage/bleeding [3]. Obstetric hemorrhage is a leading cause of fetomaternal mortality and morbidity in Sub-Saharan Africa [4]. Placenta previa has further been linked to maternal hypovolemia, anemia, and long hospital stay, as well as adverse fetal outcomes such as low birth weight, congenital abnormalities, stillbirth, and early neonatal death [5–9].

While the precise etiology of placenta previa is not known, previous studies have elucidated predictive factors such as high maternal age, twin pregnancies, previous Caesarean section, previous uterine scar, grand multiparity, malpresentation, and diabetes mellitus [5, 7–11].

The estimated global prevalence of placenta previa is 5.2 per 1000 pregnant women, although there is significant international variation, whereby the prevalence was highest among Asian studies and lower in Sub-Saharan Africa studies [12]. However, there are no studies in Tanzania which have evaluated the burden of placenta previa and its associated adverse fetomaternal outcomes. Therefore, this study aimed to determine frequency, risk factors, and adverse fetomaternal outcomes of placenta previa in Northern Tanzania. Understanding these associations would help improve early diagnosis of placenta previa and allow for better management and prevention of adverse outcomes.

## 2. Methods

### 2.1. Study Design and Setting

We conducted a retrospective cohort study using maternally linked data from the Kilimanjaro Christian Medical Centre (KCMC) medical birth registry. KCMC is located in Moshi Municipality, Tanzania. It is one of four zonal referral hospitals in the country, serving over 15 million people and performing an annual average of 4000 deliveries. The birth registry was established in 1999 as collaboration between KCMC and the registry of University of Bergen, Norway, and has been operational since 2000. Since then, all deliveries that occurred at KCMC are recorded in a computerized database system at KCMC medical birth registry.

### 2.2. Study Population and Sampling Procedure

All singleton deliveries that took place at Obstetrics and Gynecology Department of KCMC hospital from January 2000 to December 2015 with complete birth registry records were considered for analysis. Women diagnosed with placenta abruption were excluded to avoid misdiagnosis of placenta previa. In addition, women with multiple gestation pregnancies were also excluded to avoid overrepresentation of studying high risk women. The final sample was comprised of 47,686 deliveries which were analyzed.

### 2.3. Data Collection Methods and Tools

A standardized questionnaire was used to collect information for the medical birth registry. Each woman who delivered at KCMC was individually consented to interview, after which trained midwives worked through the standardized questionnaire. Women were interviewed daily, within 24 hours of delivery, or as soon as possible after recovery in case the mother had delivery complications. Information collected included maternal age, occupation, education, marital status, childhood, and present areas of residence; past medical history; last menstrual period and regularity of cycle; history of present and past smoking, drinking, or chewing tobacco use; drug, herb, and medication use; obstetric history, including first ANC visit, number of ANC visits, use of family planning, pregnancy complications, and details of labor; history of previous pregnancies, including miscarriage, stillbirth, preterm birth, fertility treatment, and mode of delivery; and sex, weight, and any medical ailments of the infant most recently delivered. The recorded information was corroborated with the antenatal cards and written medical records whenever possible. These data were entered into a computerized database system located at medical birth registry, from which they were retrieved for this study. It is worth noting that all women who deliver for the first time at KCMC are assigned with a unique mother identification number. This number is constant for all births that occur at KCMC. The same number is available to the child file; this makes it possible to link siblings with their biological mothers for subsequent births.

### 2.4. Definition of Terms

*Placenta previa* was defined as an obstetric complication characterized by placental implantation into the lower segment of the uterine wall, covering part of or the entire cervix.

*Antepartum hemorrhage* was defined as bleeding into and/or from the vaginal canal at any time from the 24th week of gestation up to the second stage of labor.

*Postpartum hemorrhage* was defined as blood loss of 500 mL or more after delivery.

*Apgar score* was defined as a measure of the physical condition of a newborn infant. The Apgar score has a maximum ten points, with two possible for each of heart rate, muscle tone, response to stimulation, and skin coloration.

### 2.5. Data Analysis

Data analysis was performed using Statistical Package for the Social Sciences (SPSS) version 20. Mean with respective standard deviation was used to summarize a normally distributed maternal age and frequencies with respective percentages were used to summarize categorical variables. Both bivariate and multivariable analysis were performed using logistic regression and adjusted odd ratios (aOR) with 95% confidence intervals for risk factors and fetomaternal outcomes associated with placenta previa were estimated in the models. A *P* value of less than 0.05 was considered statistically significant. A variable was considered as a confounder when its inclusion in the model changed the crude estimate of interest by 10%. Due to rarity of frequency of placenta previa, odds ratio was used to approximate the relative risk.

### 2.6. Ethical Considerations

Our study was approved by the Kilimanjaro Christian Medical University College Research Ethics Committee prior to its commencement. Verbal consent was obtained from all women prior to their interviews after a full explanation of the birth registry project. Research consent was not requested for this study, as the pertinent data belonged to an approved project, and confidentiality and privacy were assured as per birth registry protocol.

## 3. Results

### 3.1. Characteristics of the Study Participants

Table 1 summarizes the demographic characteristics of 47,686 singleton deliveries that were analyzed. The overall mean age was 27.48 (SD = 6.05) years. Women with placenta previa had a significantly higher mean age [29.07, SD = 6.12 (years)] than their unaffected counterparts [27.47, SD = 6.05 (years)]. Most of the women were below the age of 20 years, married, residing in urban areas, and having their ANC visit during pregnancy. An observed higher proportion of women with placenta previa were married, of younger age below 20 years, and residing in rural areas and had never attended ANC visit during pregnancy.

### 3.2. Risk Factors for Placenta Previa

Table 2 displays risk factors for placenta previa. Significant risk factors for PP after adjusting for potential confounding factors were gynecological diseases [OR 2.44; 95% CI: 1.50–3.97], use of alcohol during present pregnancy [OR 1.61; 95% CI: 1.17–2.21], grand multipara [OR 3.46; 95% CI: 1.01–11.86], and multigravida ≥5 [OR 4.85; 95% CI: 1.49–15.75]. On the other hand, antenatal care ≥4 visits [OR 0.45; 95% CI: 0.32–0.64] and maternal age ≥35 years [OR 0.56; 95% CI: 0.35–0.89] were associated with lower odds of having placenta previa. Use of family planning, previous Caesarean section, and history of previous scar had no significant association with placenta previa.

### 3.3. Maternal Outcomes Associated with Placenta Previa

Table 3 summarizes maternal outcomes associated with placenta previa. After adjustment for confounders, women with placenta previa had increased odds of APH [OR 9.21; 95% CI: 5.3–16.0] and PPH [OR 17.6; 95% CI: 8.6–36.2], hospital stay of >4 days [OR 5.62; 95% CI: 3.85–8.20], delivery by Caesarean section [OR 9.68; 95% CI: 6.66–14.1], and blood transfusion [OR 2.91; 95% CI: 1.87–4.52].

### 3.4. Fetal Outcomes Associated with Placenta Previa

Table 4 shows fetal outcomes and their associations with placenta previa. Infants delivered by mothers with placenta previa were more likely to have Apgar scores of <7 at the 1st [OR 2.68; 95% CI: 1.88–3.84], 5th [OR 3.83; 95% CI: 2.73–5.39], and 10th [OR 3.07; 95% CI: 2.08–4.52] minutes after birth, low birth weight [OR 5.62; 95% CI: 4.06–7.77], admission to neonatal intensive care unit/NICU [OR 2.53; 95% CI: 1.8–3.57], fetal malpresentation [OR 4.3; 95% CI: 2.27–8.13], stillbirth [OR 2.58; 95% CI: 1.55–4.29], and early neonatal death [OR 3.75; 95% CI: 1.15–12.3]. Congenital malformation was not associated with placenta previa.

## 4. Discussion

The prevalence of placenta previa in our study was 0.6%. Risk factors included multigravida ≥5, use of alcohol in this pregnancy, and gynecological diseases. Women with placenta previa were at increased risk for PPH, APH, need for blood transfusion, long hospital stay, and delivery by Caesarean section. Placenta previa also increased the likelihood of certain adverse pregnancy outcomes: Apgar scores ≤7 at minutes 1, 5, and 10, low birth weight, fetal malpresentation, NICU admission, and early neonatal death.

The prevalence of placenta previa in our study was similar to the prevalence of 0.7% reported in a study conducted in Pakistan by Bhutia et al. [6]. However our study shows lower prevalence than that reported by Kiondo et al. in Uganda (0.16%) [3, 6]. These differences may be explained by differences in the study design and sample size. The study conducted in Uganda had a shorter spanning time frame and had a smaller sample size, possibly leading to an underestimation of prevalence [3].

Our study found that multigravida ≥5 connoted a fivefold increase in risk of placenta previa. Similar finding was reported in studies conducted in Tanzania by Mgaya et al. and Pakistan by Raees et al. [9, 11]. The increased risk of placenta previa among multigravida women may be explained by degenerative change to the uterine vasculature, leading to underperfusion of the placenta, compensatory enlargement, and increased likelihood of implantation on the lower segment [3].

However, our study found no association between placenta previa and advanced maternal age or high parity. This differs from the findings of study conducted in Nepal by Ojha although a study conducted in Kingdom of Saudi Arabia (KSA) by Ahmed et al. also found no association between maternal age and placenta previa. Lack of association between the known risk factors with placenta previa in our study and those of others could be related to proper management of these women as a high risk group which necessitates heightened close follow-up.

Since we did not collect information on specific gynecological diseases, this odds ratio could not be compared to other studies. Sexually transmitted diseases are very common in our study area, suggesting intrauterine adhesions as a possible mechanism for impaired placental migration [10]. Past history of Caesarean section and history of uterine scar were not found to be associated with placenta previa. This contrasts with previous studies by Kiondo et al. and Anzaku and Musa [3, 13]. This may be explained by the fact that the placenta implants in a previous Caesarean section scar; it may be so deep as to prevent placental separation (placenta accreta) or penetrate through the uterine wall into surrounding structures such as the bladder (placenta percreta) which may provoke massive hemorrhage at delivery.

We found a significant association between alcohol use in the index pregnancy and placenta previa. This differs from the findings of Missouri by Aliyu et al., a discrepancy which could be explained by population differences in alcoholic intake. Alcohol use is a common practice in the study setting.

In contrast, previous studies have reported an association between smoking during pregnancy, infertility treatment, and PP [6, 14]. Unfortunately these factors are uncommon in Tanzania; thus our study may have lacked the power to assess these factors.

Our study found a significant association between placenta previa and risk of antepartum and postpartum hemorrhage: a nine times increased risk of the former and an eighteen times increased risk of the latter. Women with placenta previa had also threefold higher odds of blood transfusion and fivefold odds of prolonged hospital stay. These findings are consistent with previous studies [7, 9, 15]. The increased risk of postpartum hemorrhage in women with placenta previa may be explained by the implantation of placenta in a previous scar which may go deep preventing placental separation. This may provoke severe hemorrhage during and after delivery because the lower segment does not constrict well the maternal blood supply. This necessitates blood transfusion. Therefore, it is important that blood transfusions and the obstetric emergency care be readily available at any facility treating women with placenta previa.

Correspondingly, women with placenta previa had tenfold higher odds of Caesarean delivery [15]. This can be explained by the fact that the placenta in the lower segment obstructs engagement of the head especially for major previa. This necessitates Caesarean section and may also cause the transverse lie of the fetus.

In the present study fetal malpresentation had 3-fold higher odds of having placenta previa as compared to those with normal feta presentation of placenta previa. Our finding is consistent with previous investigators [10]. The association between placenta previa and fetal malpresentation may be explained by the fact that the placenta in the lower segment obstructs the engagement of the head; this may cause the transverse or breech lie in the womb.

Our study found that placenta previa was more common among women with <4 antenatal care visits. This may be because they were admitted earlier compared to their counterparts. Routine ultrasound examinations would be useful for early detection of women at risk of placenta previa to enhance prevention of adverse outcomes; unfortunately, the cost and maintenance of ultrasound machines hinder their utility in developing countries.

Infants born to women with placenta previa had increased odds of low birth weight, Apgar scores of <7, admission to neonatal intensive care unit, stillbirth, fetal malpresentation, and early neonatal death. This is consistent with previous studies [6, 7, 9, 16]. The possible explanation for these could be that the bleeding associated with placenta previa may lead to hypoxia, intrauterine growth restriction, and prematurity with underdeveloped organ systems. Congenital malformations were not associated significantly with placenta previa in our study.

## 5. Conclusion

The prevalence of placenta previa in our sample was consistent with past studies. Multigravidas, gynecological diseases, inadequate antenatal care, and alcohol use were key risk factors. These risk factors may be useful for screening at-risk mothers. Placenta previa was also found to connote significant risk of severe, adverse maternal and fetal outcomes. This study highlights the need for comprehensive obstetrics care to appropriately treat placenta previa and its complications.

## Figures and Tables

**Table 1:** Background characteristics of women participating in the study in Northern Tanzania (*N* = 47,686).

Characteristics	Placenta previa (*N* = 270)	No placenta previa (*N* = 47,416)	Total (*N* = 47686)
Age in years, mean (SD)	29.07 (6.12)	27.47 (6.05)	27.48 (6.05)
Age group (years)			
<20	184 (68.2)	34133 (72.5)	34317 (72.5)
20–34	30 (11.1)	6275 (13.3)	6305 (13.3)
≥35	56 (20.74)	6682 (14.2)	6738 (14.2)
Marital status			
Married	242 (90.0)	41371 (88.0)	41613 (88.0)
Not married	27 (10.0)	5657 (12.0)	5684 (12.0)
Residence			
Rural	192 (71.1)	19799 (42.0)	19991 (42.3)
Urban	69 (25.6)	25146 (53.4)	25215 (53.3)
Semiurban	9 (3.4)	2093 (4.5)	2102 (4.4)
Antenatal care in this pregnancy			
Yes	121 (44.8)	38075 (80.8)	38196 (80.6)
No	149 (55.2)	9062 (19.2)	9211 (19.4)

SD = standard deviation.

**Table 2:** Logistic regression of risk factors for placenta previa in Northern Tanzania (*N* = 47,686).

Risk factors	Total	Placenta previa	cOR (95% CI)	aOR (95% CI)
Maternal age (years)				
<20	6356	13 (0.2)	0.95 (0.53–1.72)	1.37 (0.77–2.45)
20–34	34511	74 (0.2)	1.0	1.0
≥35	6772	24 (0.4)	1.66 (1.04–2.63)	0.56 (0.35–0.89)
Gynecological diseases				
Yes	1844	22 (1.2)	0.3 (0.19–0.67)	2.44 (1.5–3.97)
No	45842	248 (0.5)	1.0	1.0
Alcohol during pregnancy				
Yes	13736	104 (38.7)	1.56 (1.21–1.99)	1.61 (1.17–2.21)
No	33814	165 (61.3)	1.0	1.0
Family planning				
Yes	29734	194 (72.4)	1.57 (1.18–2.05)	0.78 (0.46–1.31)
No	17711	74 (27.6)	1.0	1.0
History of previous scar				
Yes	2054	7 (2.6)	0.59 (0.28–1.25)	0.72 (0.30–1.72)
No	45632	263 (97.4)	1.0	1.0
Malpresentation				
Yes	657	12 (4.5)	3.38 (1.89–6.07)	4.28 (2.26–8.10)
No	46803	256 (95.5)	1.0	1.0
Number of ANC visits				
≥4	20009	67 (26.3)	0.48 (0.36–0.63)	0.45 (0.32–0.64)
<4	26762	188 (73.7)	1.0	1.0
Parity				
0	13071	39 (0.3)	1.0	1.0
1–4	18916	107 (0.6)	1.97 (1.37–2.85)	1.42 (0.48–4.16)
≥5	1060	27 (3.0)	9.91 (6.04–16.27)	3.46 (1.01–11.86)
Gravidity				
1	12118	35 (0.3)	1.0	1.0
2–4	17751	88 (0.5)	1.77 (1.2–2.62)	1.93 (0.66–5.63)
≥5	3178	50 (1.8)	6.05 (3.92–9.34)	4.85 (1.49–15.75)
Previous CS				
Yes	6958	32 (0.5)	0.84 (0.57–1.23)	0.65 (0.42–1.01)
No	26089	141 (0.6)	1.0	1.0

cOR = crude odds ratio, CS = Caesarian section, ANC = antenatal care, and aOR = adjusted odds ratio: adjusted for maternal age, gynecological diseases, alcohol during pregnancy, family planning, history of previous scar, malpresentation, number of ANC visits, parity, gravidity, and previous CS.

**Table 3:** Logistic regression for maternal outcomes associated with placenta previa (*N* = 47,686).

Maternal outcomes	Total	Placenta previa	cOR (95% CI)	aOR (95% CI)
Postpartum hemorrhage				
No	47472	254 (94.1)	1.0	1.0
Yes	214	16 (5.9)	15.02 (8.89–25.37)	17.6 (8.6–36.2)
Antepartum hemorrhage				
No	47176	487 (1.0)	1.0	1.0
Yes	510	23 (8.5)	8.97 (5.80–13.88)	9.21 (5.3–16.0)
Mode of delivery				
Vaginal delivery	3186	72 (26.7)	1.0	1.0
Caesarean delivery	15636	198 (73.3)	5.66 (4.32–7.42)	9.68 (6.66–14.08)
Need blood transfusion				
No	44586	210 (78.7)	1.0	1.0
Yes	2850	57 (21.3)	4.31 (3.21–5.79)	2.91 (1.87–4.52)
Duration of hospitalization				
≤4 days	28,676	84 (0.3)	1.0	1.0
>4 days	18599	185 (1.0)	3.42 (2.6–4.43)	5.62 (3.85–8.20)

cOR = crude odds ratio and aOR = adjusted odds ratio: adjusting for maternal age, gynecological diseases, parity, gravidity, previous CS, alcohol during pregnancy, malpresentation, and number of ANC visits.

**Table 4:** Logistic regression for fetal outcomes associated with placenta previa (*N* = 47,686).

Fetal outcomes	Total	Placenta previa	cOR (95% CI)	aOR (95% CI)
Apgar score at 1 minute				
>7	24034	72 (27.3)	1.0	1.0
≤7	23159	192 (72.7)	2.78 (2.12–3.65)	2.68 (1.88–3.84)
Apgar score at 10 minutes				
>7	42959	185 (71.2)	1.0	1.0
≤7	4027	75 (28.8)	4.39 (3.35–5.75)	3.07 (2.08–4.52)
Apgar score at 5 minutes				
>7	41526	161 (61.2)	1.0	1.0
≤7	5583	102 (38.8)	4.78 (3.73–6.14)	3.83 (2.73–5.39)
Low birth weight				
Yes	5995	130 (48.1)	6.55 (5.15–8.32)	5.62 (4.06–7.77)
No	41477	140 (51.9)	1.0	1.0
Stillbirth				
Yes	1587	28 (10.4)	3.4 (2.29–5.05)	2.58 (1.55–4.29)
No	45895	241 (89.6)	1.0	1.0
Early neonatal death				
Yes	167	5 (1.9)	5.5 (2.24–13.50)	3.75 (1.15–12.32)
No	47315	264 (98.1)	1.0	1.0
Malformation				
Yes	69	1 (0.4)	2.58 (0.36–18.68)	2.26 (0.30–16.96)
No	47531	269 (99.6)	1.0	1.0
Admission to NICU				
No	40897	183 (68)	1.0	1,0
Yes	6585	86 (32.0)	2.94 (2.28–3.81)	2.53 (1.80–3.57)

cOR = crude odds ratio, NICU = neonatal intensive care unit, and aOR = adjusted odds ratio: adjusted for maternal age, gynecological diseases, parity, gravidity, previous Caesarean section, use of alcohol during pregnancy, malpresentation, and number of ANC visits.

## Abbreviations

APH: Antepartum hemorrhage
NICU: Neonatal intensive care unit
PPH: Postpartum hemorrhage.
